# A Mosaic Genetic Screen for Genes Involved in the Early Steps of Drosophila Oogenesis

**DOI:** 10.1534/g3.112.004747

**Published:** 2013-03-01

**Authors:** Marlène Jagut, Ludivine Mihaila-Bodart, Anahi Molla-Herman, Marie-Françoise Alin, Jean-Antoine Lepesant, Jean-René Huynh

**Affiliations:** * Institut Curie, Department of Genetics and Developmental Biology (CNRS-UMR3215, Inserm-U934), 75248 Paris, Cedex 05, France; † Institut Jacques Monod, CNRS-Universite Paris Diderot, 75205 Paris, Cedex 13, France

**Keywords:** Apoptosis, Drosophila, Germ cells, Mutagenesis, Oogenesis

## Abstract

The first hours of Drosophila embryogenesis rely exclusively on maternal information stored within the egg during oogenesis. The formation of the egg chamber is thus a crucial step for the development of the future adult. It has emerged that many key developmental decisions are made during the very first stages of oogenesis. We performed a clonal genetic screen on the left arm of chromosome 2 for mutations affecting early oogenesis. During the first round of screening, we scored for defects in egg chambers morphology as an easy read-out of early abnormalities. In a second round of screening, we analyzed the localization of centrosomes and Orb protein within the oocyte, the position of the oocyte within the egg chamber, and the progression through meiosis. We have generated a collection of 71 EMS-induced mutants that affect oocyte determination, polarization, or localization. We also recovered mutants affecting the number of germline cyst divisions or the differentiation of follicle cells. Here, we describe the analysis of nine complementation groups and eight single alleles. We mapped several mutations and identified alleles of *Bicaudal-D*, *lethal(2) giant larvae*, *kuzbanian*, *GDP-mannose 4,6-dehydratase*, *tho2*, and *eiF4A*. We further report the molecular identification of two alleles of the Drosophila homolog of Che-1/AATF and demonstrate its antiapoptotic activity *in vivo*. This collection of mutants will be useful to investigate further the early steps of Drosophila oogenesis at a genetic level.

Drosophila oogenesis is a versatile model system to address many important questions of cell and developmental biology, such as stem cell regulation, cell polarization and differentiation, cell adhesion, or cell-cycle regulation (Spradling 1993). All these processes are indeed required to generate an egg chamber made of 16 germ cells, surrounded by an epithelium of somatic cells. On practical terms, the ovaries are easily accessible, yet dispensable for survival, allowing extensive manipulation. In particular, oogenesis is amenable to most of Drosophila powerful genetic tools, and it is possible to compare in the same ovary different stages of oogenesis, mutant and wild-type egg chambers side by side. Drosophila oogenesis has thus been used successfully for many genetic screens to generate collections of female sterile mutants (Schüpbach and Wieschaus 1989, 1991) or, more recently, lethal mutants using the FLP/FLP recombination target (FLP/FRT)-ovoD system (Martin *et al.* 2003; Morris *et al.* 2003; Barbosa *et al.* 2007). However, recent years have shown that key developmental decisions, such as the selection, polarization, or localization of the oocyte (the future egg cell) are made during the very early steps of oogenesis, before the arrest caused by the ovoD mutation (reviewed in Huynh and St Johnston 2004). To uncover novel genes involved during these early stages, we have performed a mosaic genetic screen by using the FLP/FRT-green fluorescent protein (GFP) system for mutations causing an early arrest of oogenesis. The morphology of the arrested egg chambers allowed us to discriminate several phenotypic classes without any staining procedures.

The Drosophila ovary is composed of 16−20 ovarioles, each of which contains a chain of progressively more and more mature egg chambers (Spradling 1993). New egg chambers are generated at the anterior of the ovariole in a region called the germarium, which has been divided into four regions according to the developmental stage of the cyst (Figure 1). Oogenesis begins in region 1, when a germline stem cell (GSC) divides asymmetrically to produce a posterior cystoblast, and a new GSC, which remains attached to the neighboring somatic cells at the anterior. The cystoblast then undergoes precisely four rounds of mitosis with incomplete cytokinesis to form a cyst of 16 germline cells, which are interconnected by stable cytoplasmic bridges called ring canals. How the number of divisions is regulated remains unknown. During these divisions, a cytoplasmic structure called the fusome anchors one pole of each mitotic spindle and therefore ensures that cells follow an invariant pattern of divisions (Lin *et al.* 1994). This leads to the formation of a symmetric cyst with two cells with four ring canals, two with three ring canals, four with two and eight with one. This invariant pattern of divisions is important because the oocyte always differentiates from one of the two cells with four ring canals, which are therefore called the pro-oocytes. How this cell is chosen remains unclear, but several lines of evidence suggest that this depends on the asymmetric segregation of the fusome during the cyst divisions, as one of the pro-oocytes always inherits more fusome than the other cell (de Cuevas and Spradling 1998). However, the link between the asymmetric inheritance of the fusome and the specification of the oocyte is still unknown.

**Figure 1:**
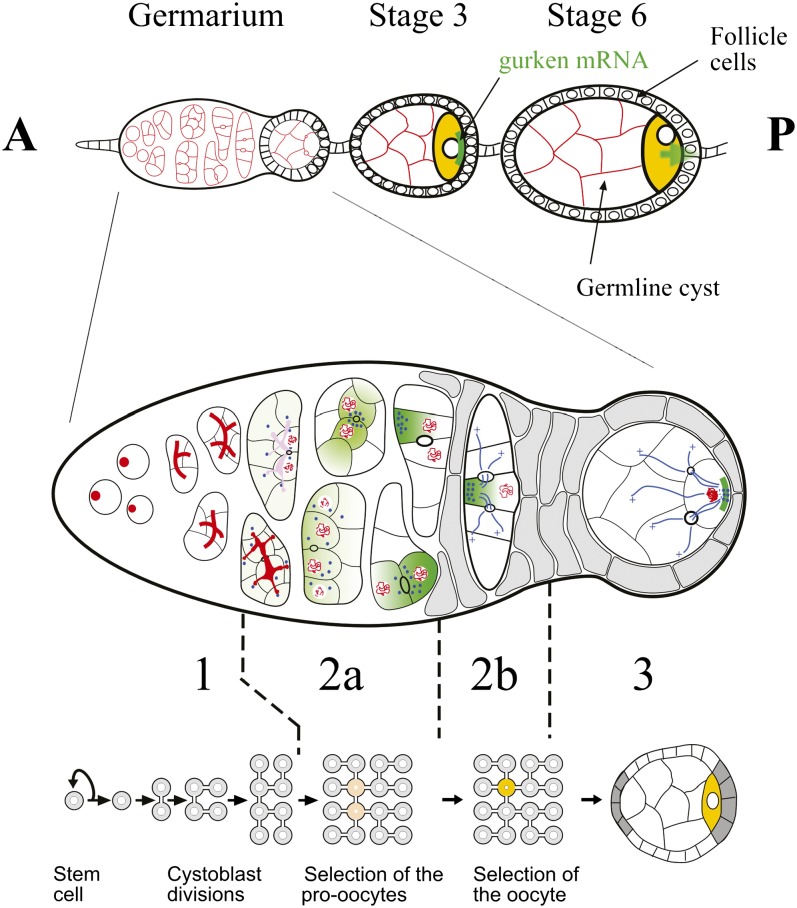
Drosophila early oogenesis. Each ovariole is made of a chain of progressively more mature egg chambers toward the posterior (P). An egg chamber comprises 16 germline cells surrounded by a monolayer of follicle cells. The egg chambers are produced at the anterior (A) of the ovariole in the germarium, which is divided into four morphological regions along the anterior-posterior axis. The germline stem cells reside at the anterior tip of the germarium (left) and divide to produce cystoblasts, which divide four more times in region 1 to produce 16 cell germline cysts that are connected by ring canals. The stem cells and cystoblasts contain a spectrosome (red circles), which develops into a branched structure called the fusome, which orients each division of the cyst. In late region 2a, the synaptonemal complex (red lines), which is a marker of meiosis, is restricted to the two cells with four ring canals (pro-oocytes, yellow). By region 2b, the oocyte has been selected and is the only cell to remain in meiosis. In region 2a, cytoplasmic proteins, mRNAs and mitochondria (green), and the centrosomes (blue circles) progressively accumulate at the anterior of the oocyte. The follicle cells (gray) also start to migrate and surround the germline cells. As the cyst moves down to region 3, the oocyte adheres strongly to the posterior follicle cells and repolarizes along its anterior-posterior axis, with the microtubule minus-ends and specific cytoplasmic components now localized at the posterior cortex (adapted from Huynh and St Johnston 2004).

Once the 16-cells cyst has formed, it enters the region 2a of the germarium. At this stage, all the cells of one cyst appear similar, but by the time it reaches region 2b, one cell will have differentiated as an oocyte. This differentiation can be followed with several types of marker (Figure 1): (1) Oocyte-specific proteins, such as Bicaudal-D (BicD), Orb, Barentsz (Btz), and Cup, and mRNAs, such as *oskar*, *BicD*, and *orb*, which first concentrate in the two pro-oocytes, and come to lie on either side of the largest ring canal which connects them (Suter *et al.* 1989; Wharton and Struhl 1989; Ephrussi *et al.* 1991; Lantz *et al.* 1994; Keyes and Spradling 1997; van Eeden *et al.* 2001). By the end of region 2a, they only accumulate in the oocyte. This accumulation depends on microtubules, the minus-end directed motor Dynein and the adaptor proteins BicD and Egalitarian (Egl) themselves (Theurkauf *et al.* 1993; Carpenter 1994; Ran *et al.* 1994; Bolívar *et al.* 2001; Navarro *et al.* 2004). (2) The centrosomes of each cell of the germline cyst appear to be inactivated after the last mitotic division and migrate along the fusome into the pro-oocytes, then into the oocyte (Mahowald and Strassheim 1970; Grieder *et al.* 2000; Bolívar *et al.* 2001). In contrast to proteins and mRNAs, this migration is not affected by microtubules depolymerizing drugs and does not depend on BicD and Egl (Bolívar *et al.* 2001). (3) The oocyte is the only cell of the cyst to remain in meiosis, and this can be followed by the formation of the synaptonemal complex as the chromosomes pair during the pachytene stage (Carpenter 1975). The restriction of meiosis to the oocyte is also resistant to colchicine treatments, but is dependent on the activity of both BicD and Egl (Huynh and St Johnston 2000). Different facets of oocyte differentiation are thus restricted to a single cell by at least three distinct pathways. It remains unclear how these pathways are selected and regulated.

By region 2b of the germarium, all these markers are restricted to only one cell of the cyst showing that the oocyte is already clearly selected. These components remain associated with the fusome remnants and therefore accumulate at the anterior of the oocyte to form a Balbiani body (Cox and Spradling 2003). When the oocyte moves through region 3, all of the components of the Balbiani body disassociate and move around the oocyte nucleus to form a tight crescent at the posterior cortex. This movement is the first sign of anterior-posterior polarity in the oocyte and is a crucial step in the maintenance of its identity. This anterior-posterior polarity requires the activity of the *par* genes, which are a conserved group of genes involved in the polarization of many cell types (Cox *et al.* 2001a,b; Huynh *et al.* 2001a,b). However, what regulate the *par* genes upstream or what the *par* genes control downstream remains unknown in the oocyte. The *par* genes could interpret an intrinsic cue left during the asymmetric divisions of the cyst. It would place the origin of the oocyte polarity as early as region 2a. Alternatively, the *par* genes could respond to an extrinsic signal sent by the follicle cells when they first contact the oocyte in region 3.

Finally, as the cyst moves down to region 3 (also called stage 1), somatic follicle cells migrate and surround the cyst to form an egg chamber. The cyst then rounds up to form a sphere with the oocyte that always lies at the posterior pole. This invariant localization of the oocyte is due to an up-regulation of the DE-cadherin in the oocyte and in the somatic cells contacting the oocyte. Therefore, the oocyte outcompetes the nurse cells for adhesion to the posterior follicle cells (Godt and Tepass 1998; González-Reyes and St Johnston 1998). This is the first *in vivo* example of a cell-sorting event that is mediated by a quantitative difference in levels of an adhesion molecule. It was further shown that the amount of DE-cadherin is regulated at the transcriptional level by Talin, an integrin-binding protein (Bécam *et al.* 2005). However, the mechanisms linking Talin to DE-cadherin transcription remain to be identified.

Although the different steps of oocyte differentiation have been recently better characterized, only a handful of genes are known to be involved in this process. Many actors are therefore still unknown at the molecular level. For example, the components that determine the oocyte fate remain unknown. The cellular machinery that restricts meiosis and the centrosomes to the oocyte is also unknown, as this process is not microtubules-dependent. Finally, although it has been shown that the oocyte is polarized as early as region 3, the origin of this polarity remains to be identified, as it could be due to an extrinsic or intrinsic signal. As many genes required for early oogenesis would also be essential for adult viability, we carried out a clonal screen in an effort to find the missing genes. We used the FLP/FRT system to generate homozygous clones of EMS-induced mutations on the chromosome arm 2L. We scored 3257 mutants lines and isolated 71 mutations. Here, we report the results of this screen and the characterization of nine complementation groups and eight single alleles. We found mutants in which the localization, determination, or polarization of the oocyte was affected. We also recovered mutants affecting the number of germline cyst divisions or the differentiation of the follicle cells. Because mutant lines were selected according to the small size and various shapes of their egg chambers, we gave names of various types of nuts to the corresponding mutant loci: *nut*, *noix*, *nuss*, *hazelnut*, *almond*, *macadamia*, *cashew*, *nutmeg*, *pecan*, *Brazil nut*, *pine nut*, *kola nut*, *gevuinanut*, *soy nut*, *pistachio*, *cacahuète*, and *coconut*. In addition, we describe in more details the molecular identification of *nutmeg*, which disrupts the novel locus CG11188. We show that CG11188 is the Drosophila homolog of Che-1/AATF, and we demonstrate the antiapoptotic activity of Che-1/AATF *in vivo*.

## Materials and Methods

### Fly stocks

The following stocks were used: the y,w; FRT40A. y,w,hs-FLP; nls-GFP,FRT40A. A Y(hs-hid) chromosome (a gift from Dr Ruth Lehmann) was used to collect virgin. The protein Hid induces cell death when it is expressed. The stock was heat-shocked two times for 2 hr in a 37° waterbath during the third larval instar and pupal stages. We checked that the few escaping males were sterile. We used *hs-FLP*; *dp,FRTG13(w+),l(2)3.112/CyO*. The *l(2)3.112* is a mutation used only as a w+ lethal marker of the second chromosome. All flies were raised at 25° unless otherwise indicated.

#### Complementation tests:

The following stocks were used for complementation tests: *BicD^R26^/CyO* (Tearle and Nusslein-Volhard 1987), *BicD^R5^* (Ran *et al.* 1994), *sop^P^/CyO* (Cramton and Laski 1994), *sop^PWR1^/CyO* (Cramton and Laski 1994), *chico^1^* (Berg and Spradling 1991), *kuz^ES24^/CyO* (Li and Baker 2001), *Gmd^H78^/CyO* (Sasamura *et al.* 2007), *y^1^w^67^c^23^*; *l(2)gl^4^/CyO*, *y+* (Mechler *et al.* 1985), *rab5^2^/CyO* (Wucherpfennig *et al.* 2003), *PI3K92E /CyO* mutation in the *dp110* gene, *PI3K21B^e02926^/CyO* (Gene Disruption Project members and Exelixis 2005) mutation in the *p60* gene, *y,w* ; *PI3K21B^e02926^,FRT40A/CyO*, *Nop60B^k05318^* (BDGP Project Members, 1994−1999) mutation in the *mini-fly* gene, *Cyc^E05206^ cn1*/CyO, *Cyc^EAR95^ cn^1^ pr^1^ bw^1^ wx^wxt^/CyO* (Knoblich *et al.* 1994), *y^1^ w^67^c^23^*; *CycE^KG00239^CyO* (Gene Disruption Project members 2001), *y^1^ w^67^c^23^Btk29A^k00206^/CyO* (Roulier *et al.* 1998), *y^1^ w^67^c^23^Pen^k14401a^ /CyO* (Török *et al.* 1993) mutation in the *importin 2* gene, *y^1^ w^67^c^23^*; *eIF-4a^k01501^/CyO* (BDGP Project Members, 1994-1999).

#### Study of the nutmeg mutants:

*Nutmeg*^21-3^ and *nutmeg*^29-3^ were identified during the screen. The two mutations failed to complement together or with *Df*(2L)Exel7027 (Bloomington-7545). *P*-element insertions in the overlapping region were tested for complementation, and the insertions PBac(RB)CG11188^e03057^ and P(EPgy2)CG11188^EY13022^ inserted in CG11188 failed to complement to the two *nutmeg* alleles. PBac(RB)CG11188^e03057^ is a lethal P-insertion in the first exon of *CG11188*, and P(EPgy2)CG11188^EY13022^ a sublethal and sterile P-insertion in the 5′UTR of *CG11188*. We recombined PBac(RB)CG11188^e03057^ on FRT40A chromosome to induce germline clones. The phenotypes observed are identical to those observed with *nutmeg*^21-3^ and *nutmeg*^29-3^.

To rescue the two nutmeg alleles, the fusion protein UAS-GFP-CG11188 (see the rescue contruct part) was recombined with the driver nanos-gal4 on the third chromosome. To test whether the phenotype of the mutant for the *CG11188* gene could be rescued by inactivating the meiotic checkpoint, we generated germline clones with the following stocks: mei41^D3^, hs-FLP ; *nutmeg*^21-3^ FRT40A, or mei41^D3^, hs-FLP ; *nutmeg*^29-3^ FRT40A in combination with mei41^D3^, hs-FLP ; (nls-GFP) FRT40A.

To test the antiapoptotic function of CG11188 *in vivo*, we used as model the polar cells in the ovaries. The following stocks were used: the driver *unpaired*-GAL4 (*upd*-GAL4) was provided by Dr Ting Xie (Stowers Institute for Medical Research, Kansas City, MO) and UAS-p35 (Neufeld *et al.* 1998). We compared the number of polar cells between flies: (1) white 1118, (2) *upd*-GAL4/+ ; UASp35/+, and (3) *upd*-GAL4/+ ; UASp-CG11188-GFP/+

### Mutagenesis

**Figure 2:**
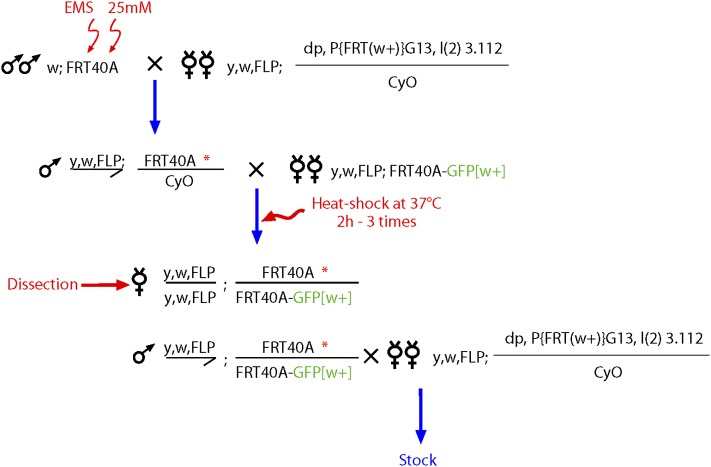
Crossing scheme for isolating mutants affecting early oogenesis on 2L. An asterisk indicates the EMS-treated chromosome. Males carrying an FRT40A chromosome were mutagenized with EMS and crossed in mass to females carrying the FLP (Figure 2) and a lethal w+ mutation balanced over CyO. Each mutation is recovered over a balancer chromosome. In the progeny, males were crossed individually to five females to generate clones with the FLP/FRT system on the left arm of the chromosome 2. Larvae were heat-shocked at 37° for 2 hr on 3 consecutive days. In the progeny, ovaries of five females were dissected for each cross. Interesting mutations were recovered through the brothers, which were backcrossed to the same lethal w+ mutation balanced over CyO.

**Figure 3:**
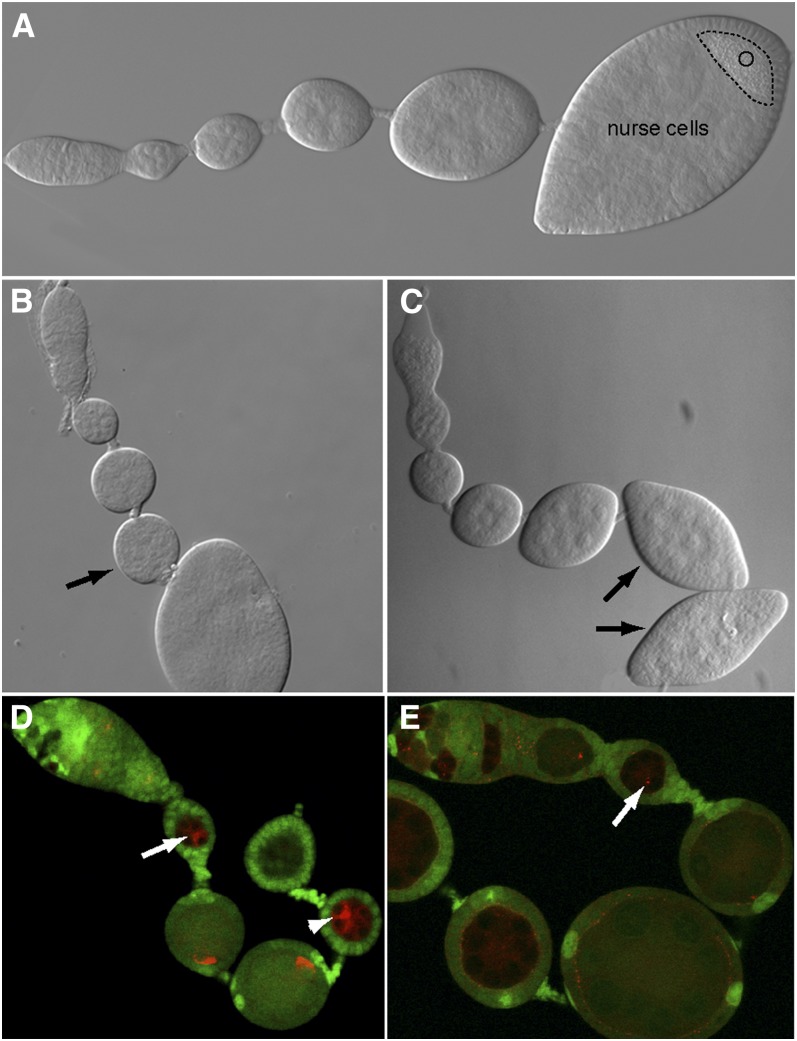
Overview of the screen. (A−C) Ovarioles observed by Nomarski microscopy. (A) Wild-type ovariole. O, oocyte (B) Mosaic ovariole that contains some arrested egg chambers (black arrow). The mutant egg chamber is smaller than the younger one that precedes it. (C) Egg chamber in which the oocyte is absent and replaced by a nurse cell. In this case, the egg chamber takes the characteristic shape of a lozenge (black arrows). (D−E) Mutant germline clones labeled by the lack of GFP (green). Their development is stopped at stage 2. (D) Orb protein (red) remains at the anterior of the oocyte (arrow). (E) Centrosomes (red), stained with an anti-γ-tubulin antibody, remain at the anterior of the oocyte (arrow).

The screen was performed for the 2L chromosome. y,w; FRT40A males were starved for 6 hr before being exposed to 30 mM EMS (Sigma-Aldrich; M0880) in 1% sucrose for 18−24 hr to induce an average of one lethal hit per chromosome arm. The number of lethal hit was estimated by monitoring the number of X-linked lethals. Mutagenised males were mated in mass with *hsFLP*; *dp,FRTG13(w+),l(2)3.112/CyO* virgin females. Single *y,w*, *hs-FLP/Y*; FRT40A*/CyO (the asterisk indicates the mutagenised chromosome) were mated with five *y,w,hs-FLP*; *nls-GFP,FRT40A* virgin females. The progeny were heat-shocked three times for 2 hr in a 37° waterbath during the third larval instar and pupal stages (Figure 2). Ovaries from three to five females of genotype y,w,hs-FLP;FRT 40A*/FRT40A, *nls-GFP* were dissected and screened for defects of egg chambers morphology using a binocular (Figure 3). If a phenotype was observed, *y,w,hs-FLP/Y*; FRT40A*/nls-GFP,FRT40A males were mated with *hsFLP*; *dp,FRTG13(w+),l(2)3.112/CyO* virgin females and balanced stocks were established. Mutants identified during the primary screen were further analyzed with a confocal (Leica SP5) or apotome (Zeiss) microscopes.

### Staining procedure

Antibody staining and Hoechst staining were performed according to standard protocols (*i.e.*, dissection in phosphate-buffered saline 1×, fixation 20 min in 4% paraformaldehyde, staining in PBS + 0.1% Triton (PBT). The antibodies used were as follows: mouse anti-Orb at 1/250 [6H8 and 4H8 from Developmental Studies Hybridoma Bank, University of Iowa (Lantz *et al.* 1994)], mouse anti-γ-Tubulin (Sigma-Aldrich) at 1/100 or rabbit anti-CP309 at 1/500 (Kawaguchi and Zheng 2004) to stain centrosomes, rabbit anti-C(3)G at 1/1000 (Hong *et al.* 2003), rhodamin-phalloidin at 1/500 (Molecular Probes), rat anti-DE-Cadherin (D-CAD2, DHSB) at 1/20, rabbit anti-Wcd at 1/500 (Fichelson *et al.* 2009), rabbit polyclonal anti- γ-His2av at 1/500 (provided by Dr Kim McKim, Waksman Institute, Piscataway, NJ), and mouse monoclonal anti-Fasciclin III (1:30; Developmental Studies Hybridoma Bank). Ovaries were mounted in Citifluor.

### Complementation tests

All the mutant lines, which were kept after the two rounds of screen, were used in complementation crosses. The criteria for a lack of complementation were the lethality or the sterility of *trans*-heterozygous animals. With these criteria, we obtained 10 distinct complementation groups formed by two to five alleles respectively. We also obtained a group of 19 mutants that produce, during complementation crosses, *trans*-heterozygous animals smaller and completely sterile. We concluded that these mutants interact genetically. Mutant lines showing specific phenotypes were crossed to known mutants on the 2L giving similar phenotypes. Our mutants were crossed with *BicD*, *sop*, *chico*, *kuz*, *gmd*, *l(2)gl*, *rab5*, *p60*, *cycE*, *Btk29A*, *Pen*.

### Deficiency mapping

All lethal complementation groups and also interesting lethal single alleles were crossed to deficiencies of the Exelixis collection for the 2L. We also used Bloomington deficiencies to cover gaps in the Exelixis kit. We tested for lethality over the deficiencies. When a deficiency did not complement with one mutant of a complementation group, we verified the result with the other mutants of the group. For some mutants, we were able to identify a region in which the mutation could be localized by deficiency mapping. Available mutants in the region were then crossed to our mutant. Using this method, we identified the *pecan* mutant as a novel allele of *eiF4A*, 2 alleles of *GDP-mannose 4,6-dehydradtase* (*Gmd*), 1 allele of the metalloprotease *kuzbanian* (*kuz*), and 2 alleles of CG11188.

### Alleles sequencing

Genomic DNA was extracted from homozygous larvae. Homozygous larvae were identified by the absence of GFP expression of the CyO balancer. We used this method to sequence candidate genes in the region of the *macadamia* mutant, the two *nutmeg* alleles and three mutants of class III. For the *macadamia* mutants, we identified amino acid substitution in several alleles of the *tho2* gene. For one the two *nutmeg* alleles, we identified an amino acid substitution in *CG11188* gene.

### Rescue construct and GFP-tagged protein for CG11188

A polymerase chain reaction fragment corresponding to the whole *CG11188* cDNA sequence was amplified from the DGRC cDNA clone no. RE30678 using primers 5′-CACCATGCTGCGCAAGTCAAAG-3′ and 5′-GACAAACAATGATTTGTACAAC-3′ and cloned into the pENTR/D-TOPO Gateway entry vector using the pENTR directional TOPO cloning kit (Invitrogen). The *CG11188* cDNA was then transferred to Drosophila transgenic expression vectors by LR recombination using the Gateway LR clonase II enzyme mix (Invitrogen). Destination vectors pPWG was obtained from Terence Murphy’s laboratory Drosophila Gateway Vector Collection at Carnegie Institution (http://www.ciwemb.edu/labs/murphy/Gateway%20vectors.html). The resulting transgenic construct consists of eGFP N-terminal tagged (GFP::CG11188) CG11188 fusion protein, under the control of the UASp promoter, allowing GAL4-driven somatic and female germline expression. Transgenic lines were generated by standard methods.

### Sequence comparison

We obtained protein sequences thanks to Genebank database: *Drosophila melanogaster* CG11188 (AAF52427), *Homo sapiens* Che-1 (AAH00591), *Mus musculus* AATF (NP_062790), *Rattus norvegicus* AATF (AAH78769), and *Xenopus laevis* AATF (NP_001167477). Similarities between CG11188 and Che-1/AATF related proteins were analyzed using Clustal W and Mac Vector.

## Results

### Generating mutants and primary screen

We performed a mosaic genetic screen by using the FLP/FRT system to induce homozygous mutant clones in heterozygous individuals (Chou and Perrimon 1992; Xu and Rubin 1993). Clones are obtained by the recombination of two FRT sequences after the heat shock− inducible expression of the Flipase (FLP) recombinase. As a heat shock promoter drives the expression of the FLP, clones can be induced both in the germline and somatic cells. We mutagenized males carrying an FRT40A chromosome with ethyl methyl sulfonate (EMS) and crossed them in mass to females carrying the FLP (Figure 2). Each mutation is recovered over a balancer chromosome. In the progeny, males were crossed individually to five females to generate clones with the FLP/FRT system on the left arm of the chromosome 2. The crossing scheme is detailed in Figure 2. During the primary screen, we dissected five females for each line under a dissection scope and screened for egg chambers with a developmental delay, early arrest, or with morphological defects, without any fixation or staining procedure. These defects were easy to detect as mutant egg chambers can be compared with the wild-type ones in the same ovariole. In wild-type ovarioles, egg chambers increase steadily in size toward the posterior of the ovary (Figure 3A). A precocious arrest in development generates an egg chamber obviously smaller than the preceding younger and wild-type egg chamber (Figure 3B). Similarly, abnormalities in the shape of egg chambers can reveal developmental defects. For instance, egg chambers that contain 16 nurse cells and no oocyte have a specific lozenge shape, as posterior follicular cells adopt by default the anterior identity. This phenotype is observed in *BicD* and *egl* mutants (Figure 3C). The localization of the oocyte can also be monitored with a low magnification scope thanks to the autofluorescence of granules of vitellus, which accumulate only within the oocyte later during oogenesis. Thus, we used the morphology of stage 3 to 6 egg chambers, before the arrest caused by ovoD mutation, as a read-out of defects originating in the germarium. This strategy has the additional advantage to eliminate most cell-lethal mutations, which would not allow the formation of a 16-cell cyst able to exit the germarium. In this first round of screening, we dissected 3257 independent lines among which we kept 225 mutants for further analysis in a secondary screen.

### Secondary screen: characterization of the phenotype of early oogenesis mutants

During the second round of screening, we characterized in more detail each of the 225 mutants kept in the first round. Homozygous mutant clones were identified by the absence of a nuclear-GFP and mosaic ovaries were stained for four different markers: (1) we analyzed the determination and polarization of the oocyte by immunostainings for Orb and the centrosomes (Figure 3, D and E); (2) we could also identify the oocyte by looking at the condensation of its DNA into a karyosome; and (3) we checked the progression through meiosis by following the formation and restriction of the synaptonemal complex using an anti-C(3)G antibody. As described in the introduction, those markers are restricted into the oocyte along at least three different pathways. Although most mutants caused a similar early arrest of oogenesis, these markers allowed us to discriminate several categories of phenotypes. After this secondary screen, 71 mutant lines showing an interesting and penetrant phenotype were kept, which is about 32% of the lines remaining after the first round of screen and 2.2% of the lines initially scored. We classified these mutants into eight phenotypic classes that are presented in Table 1 and explained in more details below (Table 2).

**Table 1:** Classification of mutants generated in the screen

Phenotypic Class	Number of Mutants	Number of Single Alleles	Number of Alleles in Complementation Groups (Number of Groups)
Class I: Oocyte determination	2	/	2 (1)
Class II: Early arrests before oocyte repolarization	32	15	17 (6)
Class III: Early arrests without defects (class A)	6	6	/
Class III: Early arrests without defects (class B)	19	19	Genetic interaction between all mutants
Class IV: Follicular proliferation	8	4	4 (2)
Class V: Cyst division	1	1	/
Class VI: Oocyte localization	1	1	/
Class VII: Oocyte growth	1	1	/
Class VIII: Orb posterior localization	1	1	/
Total	71	48	23 (9)

**Table 2:** 2L complementation groups obtained in the screen

Mutant Class	Locus Name	Number of Alleles	Allele Names	Mutation Localization	Gene	Lethality
Class I: Oocyte determination	*almond*	2	14.4/ 28.3	36C9	*BicD*	Lethal
Class II: development arrests before oocyte repolarization	*macadamia*	5	19.2/ 26.3	22D1	*tho2*	Lethal
			27.7/ 46.3			
			47.1			
	*cashew*	4	20.7/ 26.5	?	*?*	Lethal
			28.5/ 29.9			
	*nutmeg*	2	21.3/ 29.3	27A1	*?*	Lethal
	*nut*	2	16.1/ 29.4	?	*?*	Lethal
	*noix*	2	16.5/ 20.6	?	*?*	Lethal
	*nuss*	2	29.5/ 43.7	?	*?*	Lethal
	*pecan*	1	19.6	26B2	*eIF4A*	Lethal
Class III: early arrests without defects	class A	6	/	/	*/*	Lethal
	class B	19	/	/	*/*	Lethal (15) *minute* phenotype and sterility (4)
Class IV: follicular proliferation	*Brazil nut*	2	10.8/ 17.6	25B5	*Gmd*	Lethal
	*pine nut*	2	43.1/ 46.2	?	*?*	Lethal
	*Kola nut*	1	43.4	21A5	*l(2)gl*	Lethal
Class IV: follicular proliferation	*gevuinanut*	1	46.3	?	*?*	Lethal
	*soy nut*	1	46.10	34C6	*kuz*	Lethal
Class V: cyst divisions	*hazelnut*	1	30.1	28E3-29C1	*?*	Lethal
	*walnut*	2	37.5/ 40.2	25A7-25B1	*?*	Lethal
Class VI: oocyte localization	*pistachio*	1	32.1	26F1	*?*	Lethal
Class VII: oocyte growth	*cacahuète*	1	48.1	?	*?*	Lethal
Class VII: Orb posterior localization	*coconut*	1	18.2	21B5-21B8	*?*	Sublethal
						Female serile
						Male sterile

### Mapping and complementation groups

To determine how many genes were represented among the 71 mutants, we placed these lines into complementation groups. Besides testing *trans*-heterozygous flies for lethality, we also tested their fertility, as some of our mutants were not lethal. We first crossed mutants of the same phenotypic class between each other. We found nine groups of complementation formed by 23 mutants (see Table 1). In addition, we found a group of 19 genes interacting genetically. These 19 mutants showed growth defects during oogenesis, and gave rise to viable *trans*-heterozygous flies, which were smaller than wild type and completely sterile (Figure 5D). We then crossed all complementation groups to all remaining mutant lines. We did not identify any additional allele during this step, and we were thus left with 29 single-allele mutant lines. The nine complementation groups and the eight most interesting single alleles were mapped by crossing to the Exelixis deficiency kit for chromosome 2L (Parks *et al.* 2004), additional deficiencies covering the main gaps of the kit (available at DrosDel and Bloomington) and to available mutants within these deficiencies. During these tests, we identified novel alleles of genes known to affect early oogenesis: two alleles of *BicD* and one allele of *lgl*. We also identified one allele of *eIF4A*, 2 alleles of *GDP-mannose 4,6-dehydratase* (*Gmd*), and one allele of the metalloprotease *kuzbanian (kuz)*.

### Class I: Mutants affecting the identity of the oocyte

We found two mutant lines, which gave rise to lozenge shape egg chambers at a high frequency. These two lines were lethal when crossed to each other and formed the *almond* group of complementation. *almond* mutant egg chambers comprised 16 polyploid nurse cells and no oocyte as revealed by DNA staining. Although Orb failed to accumulate in any specific cell of the cyst, centrosomes were in contrast restricted at the anterior of a single cell. It showed that the oocyte was correctly selected but eventually differentiated as a nurse cell (data not shown). Egg chambers mutant for null alleles *Bicaudal-D*, which is on chromosome 2L, show an identical phenotype (Huynh and St Johnston 2000; Bolívar *et al.* 2001). Both alleles of *almond* were indeed lethal over *BicD^r5^* and are thus two novel alleles of *BicD*, as strong as the null allele *BicD^r5^* (Ran *et al.* 1994). This result showed that we were able to generate and identify two alleles of *Bicaudal-D*, which validated our screening strategy.

### Class II: Mutants arresting oogenesis before oocyte repolarization

**Figure 4:**
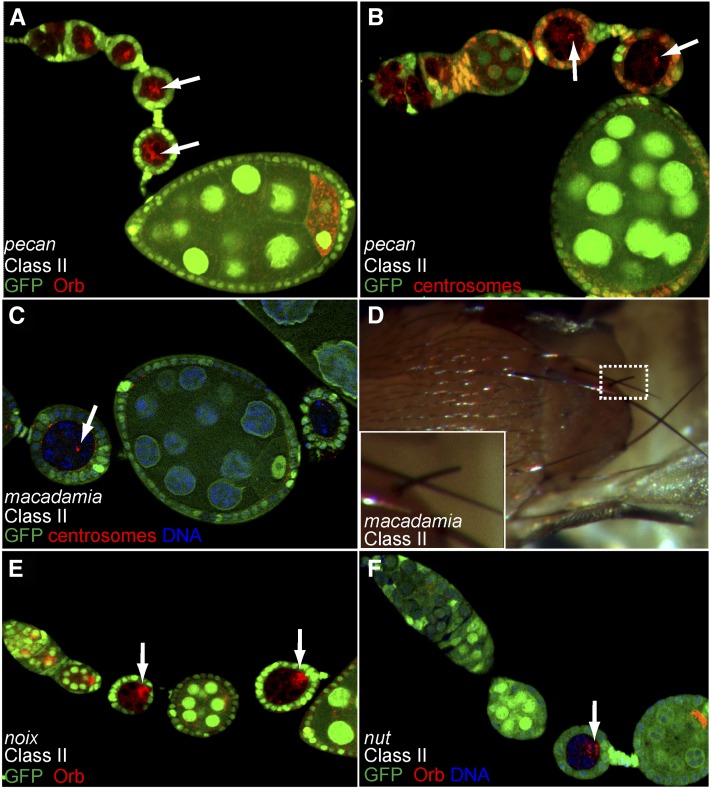
Phenotypic description of mutants of Class II. (A−F) Mutant germline clones labeled by the lack of GFP (green). (A, B) Arrested *pecan* germline clones (arrows), in which the oocyte is not correctly polarized: Orb (red) (A) and centrosomes (red) (B) remain at the anterior of the oocyte. (C) *macadamia* germline clones stained for γ-tubulin (red). Centrosomes (arrow) remain at the anterior of the oocyte. *macadamia* germline clones rapidly degenerate. (D) Flies with somatic clones mutant for *macadamia*, often present short bristles (inset). (E) The development of germline clones mutant for *noix* is delayed (arrows). Orb protein (red) can be found at the anterior of the oocyte, migrating (arrow) to the posterior and in some cases at the posterior of the oocyte. (F) The development of germline clones mutant for *nut* is delayed (arrow). Orb (red) migration to the posterior is mainly delayed (arrow) in mutant germline clones.

In this category, we classified 32 lines in which mutant egg chambers stopped their development around stage 2 to 4 of oogenesis. Orb and the centrosomes were correctly restricted to one cell in each mutant cyst but failed to localize to the posterior pole of the oocyte in most cases (Figure 4, A and B). In this class, 17 lines were placed into six complementation groups: *macadamia*, *cashew*, *nut*, *noix*, *nuss*, and *nutmeg*. The remaining 15 lines are single alleles and will not be discussed further, with the exception of the *pecan* locus, which induced a very penetrant and strong phenotype.

The *macadamia* group of complementation is made of five alleles, which induced an arrest of oogenesis at stage 3, with Orb and the centrosomes at the anterior of the oocyte (Figure 4C and data not shown). This arrest was rapidly followed by the degeneration of mutant egg chambers. *macadamia* alleles were lethal over several deficiencies, restricting the locus to the genomic region 22D1. We then sequenced several genes in the region and found for two alleles of three tested, two independent amino-acid substitutions in exon 6 of *tho2* (aa 499 G into V and aa 470 Y into H). The five *macadamia* alleles are the first known alleles of *tho2*.

We isolated four alleles of *cashew*, *20-7*, *26-5*, *28-5*, and *29-9*, in which mutant egg chambers arrested their development at stage 3. The localization of Orb was slightly variable: in the majority of mutant cysts, Orb remained at the anterior, but Orb could also be seen on the side of the oocyte nucleus and even at the posterior pole of the oocyte in some cases. In contrast, the centrosomes always remained at the anterior of the oocyte (data not shown). All 4 alleles complemented all the deficiencies we tested, and we could thus not map the locus to any genomic region.

*nut*, *noix*, and *nuss* groups are each made of two alleles and displayed very similar phenotypes. Mutant germline cysts were arrested between stage 3 and 5, and the localization of Orb was also variable, as Orb could be found at the anterior of the oocyte, migrating around the oocyte nucleus or at the posterior pole (Figure 4, E and F). The frequency of each case varied with the allele. We interpreted this phenotype as a delay in Orb restriction rather than a failure to polarize the oocyte. All six mutant lines complemented all the deficiencies we tested, and we could not map any of the loci to a specific genomic region.

The two alleles of *nutmeg* displayed a penetrant arrest at stage 3 of oogenesis with Orb and the centrosomes remaining strictly at the anterior of mutant oocytes (Figure 9D). Both alleles were lethal over several deficiencies and we could map the mutations to the *CG11188* locus as described in more details below. The *pecan* single allele gave an identical phenotype to *nutmeg* with the same penetrance (Figure 4, A and B). Deficiency mapping localized the mutation to the 26B2 region and further complementation tests showed that *pecan* was lethal over mutations in the *eukaryotic initiation factor 4A* (*eIF4A*) gene, which encodes an ATP-dependant RNA helicase, essential for translation initiation. This gene has been described as being required cell-autonomously for growth and cell division (Galloni and Edgar 1999).

### Class III: Early arrested egg chambers with correctly determined and polarized oocytes

**Figure 5:**
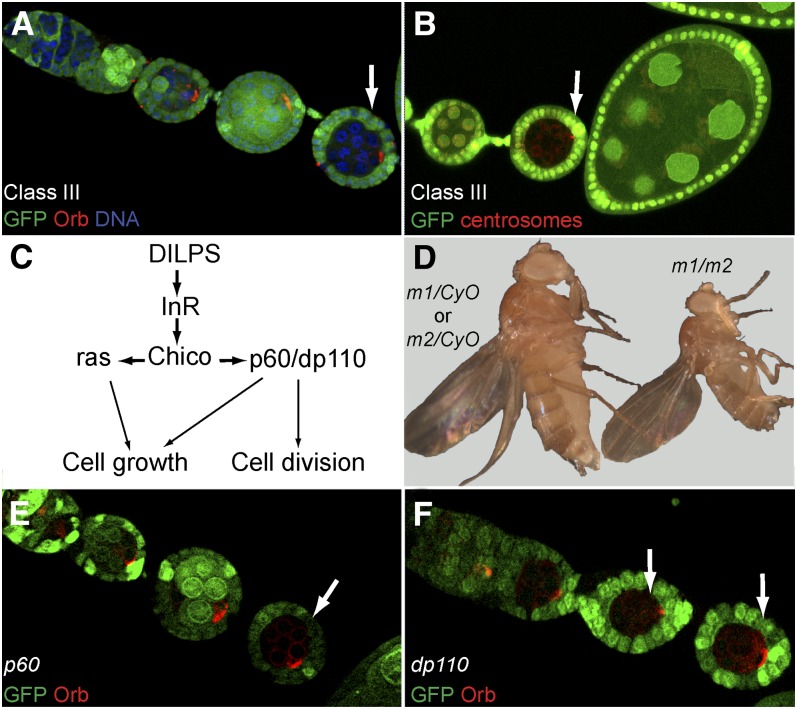
Phenotypic description of the mutants of Class III and mutants of the insulin pathway. (A−F) Mutant germline clones labeled by the lack of GFP (green). (A, B) Class III mutant germline clones stop their development very early between stages 2 and 3. (A) Orb protein (red) is correctly localized at the posterior of the oocyte (arrow). (B) Centrosomes (red) are also at the posterior of the oocyte (arrow). (C) Simplified scheme of the Drosophila insulin pathway. (D) *trans*-heterozygous flies obtained during complementation tests are reduced in size. *m1* and *m2* are any two different alleles of these 19 mutants. (E) *p60* mutant germline clones. (F) *dp110* mutant germline clones (E, F) Orb protein (red) is correctly localized at the posterior of the oocyte (arrows) as in wild type in both *p60* and *dp110* mutant germline clones.

In this phenotypic class, germline mutant egg chambers stopped their development between stage 2 and 4. Despite their small size, Orb and the centrosomes were correctly localized at the posterior pole of the oocyte (Figure 5, A and B). The karyosome was properly condensed and the oocyte was maintained in meiosis (data not shown). The only obvious defect was thus a failure to grow. A total of 25 mutant lines were categorized in this class and we further distinguished two subgroups, a first group of 19 alleles strongly interacting genetically and a second group of six single alleles that will not be described further.

In the first group of mutants, any *trans*-heterozygous combinations of the 19 alleles gave rise to viable but tiny flies, whose ovaries failed to develop and were thus sterile (Figure 5D). Among the 19 mutant lines, four were viable and also sterile. The small and sterile *trans*-heterozygous flies are very similar to *minute* flies observed when ribosome biogenesis or the insulin pathway are affected. To test for potential genetic interaction with the ribosome biogenesis pathway, we used mutant alleles of *minifly* (also called *nop60B*), a Drosophila homolog of Dyskerin, which is essential for ribosomal RNA maturation (Giordano *et al.* 1999). Although homozygous *minifly^1^* female are reduced in size and almost sterile, all 19 *trans*-heterozygous progeny were of normal size and fertility. We thus did not detect genetic interaction with this member of the ribosome biogenesis pathway.

Activation of the insulin pathway leads to the recruitment of Chico (a *Drosophila* homolog of vertebrate insulin receptor substrate-4) by the Insulin Receptor (InR), which in turn recruits the phosphatidyl-inositol-3-kinase Dp110, through the adaptor protein p60 (Goberdhan and Wilson 2003; Oldham and Hafen 2003; Hafen 2004). This cascade then triggers the activation of at least two downstream effectors: PKB/Akt and Grb2/Drk (Figure 5C). A recent study showed that the input of the insulin pathway on GSC proliferation, cyst growth, and vitellogenesis is entirely mediated by phosphatidyl-inositol-3-kinase in Drosophila (Hsu *et al.* 2008). We further found that germline clones mutant for *p60* or *dp110* arrested oogenesis early and showed an identical phenotype to our Class III mutants (Figure 5, E and F). We thus tested for genetic interactions between *chico^1^*, *p60*, *dp110*, and our Class III mutants. We found that 17 lines of the first group of mutants gave rise to small and sterile flies when crossed to *chico^1^*. Surprisingly, none of the mutant lines interacted with *p60* or *dp110*. One line of the second group of mutants did, however, interact genetically with *p60*. *chico* and *p60* are localized on chromosome arm 2L, it is thus possible that some of our mutations are alleles of *chico* or *p60*. We sequenced three lines and we did not find any mutation in the coding sequence of *chico*, indicating that at least three of our mutants are probably not alleles of *chico*. Furthermore, the *chico* locus is approximately 4 kb, and the probability that this locus was hit 17 times independently is low. However, we cannot formally exclude that they are alleles of *chico* until we mapped them meiotically.

### Class IV: Egg chambers encapsulation defects

**Figure 6:**
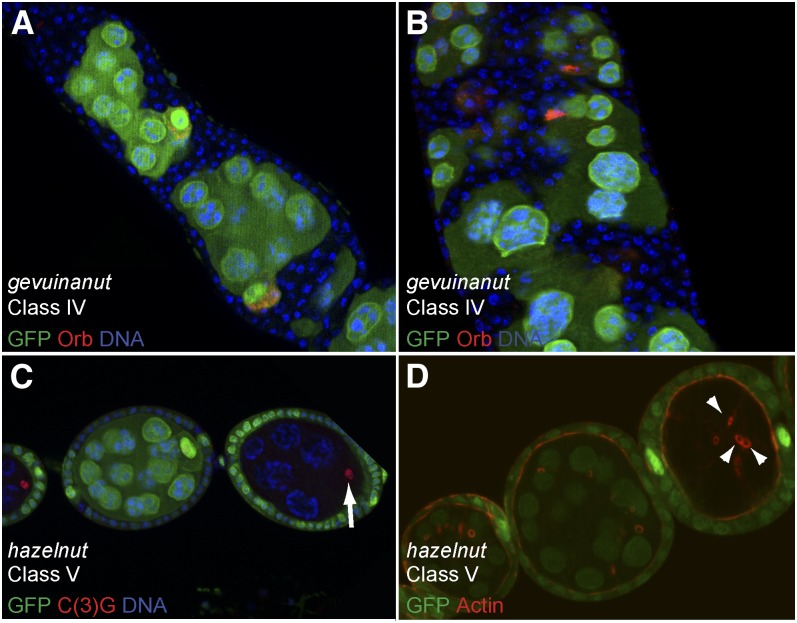
Phenotypic description of mutants of Classes IV and V. (A−D) Follicular and germline clones labeled by the lack of GFP (green). (A, B) All follicle cells are mutant for *gevuinanut*. Germline cells are wild type (green) and stained for Orb (red), which labels the oocyte. Mutant follicle cells overproliferate (A) and trigger encapsulation defects. In some cases, follicular tumor invades in between germline cells (B). (C, D) *hazelnut* mutant germline clones are made of only eight cells. (C) Synaptonemal complex (red) is restricted to one cell (arrow), suggesting that the oocyte is correctly determined. (D) The oocyte is connected to the nurse cells by only three ring canals (arrows) as shown by actin staining (red).

In this class, mutant egg chambers are characterized by the encapsulation of several germline cysts within a single egg chamber. The follicular epithelium also was disorganized as several layers of follicle cells often surrounded those multi-cyst egg chambers (Figure 6, A and B). Eight mutants were classified in this group and in all cases the phenotype was follicle cells-dependent. Four mutants made up two complementation groups, *Brazil nut* and *pine nut*, and the remaining four mutants were single alleles.

The encapsulation defects and excess of follicle cells are reminiscent of phenotypes induced by mutations affecting the Notch pathway. Germline clones mutant for the ligand Delta, or follicle cell clones mutant for the receptor Notch lead to the formation of similar fused egg chambers with multiple layers of follicle cells (López-Schier and St Johnston 2001). In these mutant conditions, follicle cells fail to differentiate and, in particular, fail to switch from the mitotic cycle to a specialized endocycle in which the M phase is skipped. This can be followed by DNA staining, as undifferentiated and proliferating cells have small nuclei, whereas endocycling cells increase their DNA contents from stage 6 onward. We found for the eight lines in this class that homozygous mutant follicle cells had small nuclei beyond stage 6 egg chambers (Figure 6, A and B). We thus performed complementation tests with candidate genes of the Notch pathway localized on chromosome arm 2L. We found that the two alleles of *Brazil nut* failed to complement mutations in the gene *GDP-mannose 4,6-dehydratase* (*Gmd*). Gmd is required to produce O-fucose, which is necessary for the O-fucosylation of Notch. This post-translational modification transforms Notch in a substrate for Fringe (Okajima *et al.* 2005; Vodovar and Schweisguth 2008). In addition, we found that the single allele *soy nut* did not complement mutations in *kuzbanian* (*kuz*). Kuzbanian is an ADAM (A Disintegrin And Metalloprotease) protein, which is the homolog of the vertebrate ADAM10 (Rooke *et al.* 1996). It plays a critical role during the proteolytic conversion of Notch required to activate the pathway (Pan and Rubin 1997; Sotillos *et al.* 1997).

Mutations in tumor suppressor genes such as *disc large* (*dlg*) or *lethal(2) giant larvae* (*lgl*) also induce an overproliferation of follicle cells (Goode and Perrimon 1997; Bilder *et al.* 2000). In addition, homozygous mutant cells were described to invade the germline cells by migrating between the nurse cells (Goode and Perrimon 1997). We found by complementation tests that the single allele *Kola nut* was a novel allele of *lgl*. This mutant showed a dramatic overproliferation of follicle cells, but the invasive behavior was mild (data not shown). In contrast, follicle cells mutant for *gevuinanut*, another class IV single allele, showed a massive invasion of germline cells (Figure 6, A and B). This allele complemented all the deficiencies tested and thus remains unlocalized on 2L.

### Class V: Mutant affecting the divisions of the germline cyst

Wild-type germline cysts always go through four rounds of oriented mitosis and thus contain two cells with four ring canals, two with three, four with two, and eight with one (de Cuevas *et al.* 1997). We found one single allele, *hazelnut*, in which a subset of homozygous mutant germline cysts was made of only eight germ cells (Figure 6C). A staining for actin allowed us to count the number of ring canals, and we found that mutant oocytes had only three ring canals (Figure 6D), indicating that mutant cysts underwent only three divisions instead of four. The oocyte was correctly determined as shown by the restriction of the SC to one cell at the posterior of the egg chamber (Figure 6C). The other subset of *hazelnut* mutant cysts did four divisions but arrested oogenesis early and exhibited polarity defects similar to Class II mutants (data not shown). Only a few genes are known to regulate the number of germline cyst divisions. Overexpression of *string/cdc25* inhibits the fourth division to produce cysts with 8 cells, 50% of which lack an oocyte. The same phenotype is observed in loss-of-function of *tribbles*, which is a negative regulator of *string* (Mata *et al.* 2000). Loss of function of *cyclinE*, *half-pint* and *ovarian tumor* (*otu*) also produce eight cells-cysts (King and Storto 1988; Storto and King 1988; Lilly and Spradling 1996; Van Buskirk and Schüpbach 2002). We mapped *hazelnut* lethality to overlapping deficiencies in the 28E3-29C1 region; however, no candidate gene related to the genes mentioned previously was obvious to sequence.

### Class VI: Mutant inducing a mislocalization of the oocyte

**Figure 7:**
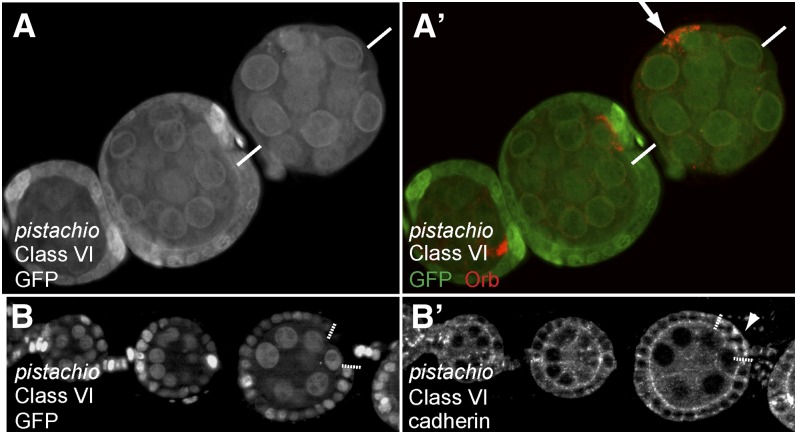
Phenotypic description of the mutants of Class VI. (A−B) Mutant follicular clones labeled by the lack of GFP (green). (A, A′) Ovariole stained for the Orb protein (red). The oocyte (arrow) is mislocalized to one side of the egg chamber, when all follicle cells are homozygous mutant for *pistachio*. The two white bars represent the anterior-posterior axis as determined by the stalk cells. (B) *pistachio* homozygous mutant follicle cells (white dotted lines). (B′) DE-cadherin channel is shown on its own. DE-cadherin is mislocalized in the cytoplasm and basal side of follicle cells mutant for *pistachio* (arrowhead), instead of being restricted to the apical cortex.

We isolated one single allele, called *pistachio*, in which the oocyte was localized on the side of the egg chamber, instead of being at the posterior (Figure 7, A and A′). During the second round of screen, we found that this phenotype was somatic-dependent, as mislocalization of the oocyte could only be observed when large clones of homozygous mutant follicle cells were induced. In addition, we found that germline clones mutant for *pistachio* arrested oogenesis early at stage 3. In these germline mutant egg chambers, the oocyte was correctly determined, polarized, and localized at the posterior pole (data not shown). The position of the oocyte is thought to depend mainly on greater expression of DE-cadherin in the oocyte and posterior follicle cells. Germline and follicle cells clones mutant for DE-Cadherin induce a mislocalization of the oocyte (Godt and Tepass 1998; González-Reyes and St Johnston 1998). We thus analyzed the expression and localization of DE-cadherin in follicle cells mutant for *pistachio*. We found that mutant cells seemed to express greater levels of DE-cadherin and also that the protein was mislocalized in the cytoplasm and basal side of follicle cells, instead of being restricted to the apical cortex (Figure 7, B and B′). Whether these expression and/or localization defects of DE-cadherin are sufficient to explain the mislocalization of the oocyte, remains to be determined. Deficiency mapping localized *pistachio* lethality to region 26F1. However, no candidate gene appeared obvious to sequence in the region.

### Class VII: Mutant affecting the growth of the oocyte

**Figure 8:**
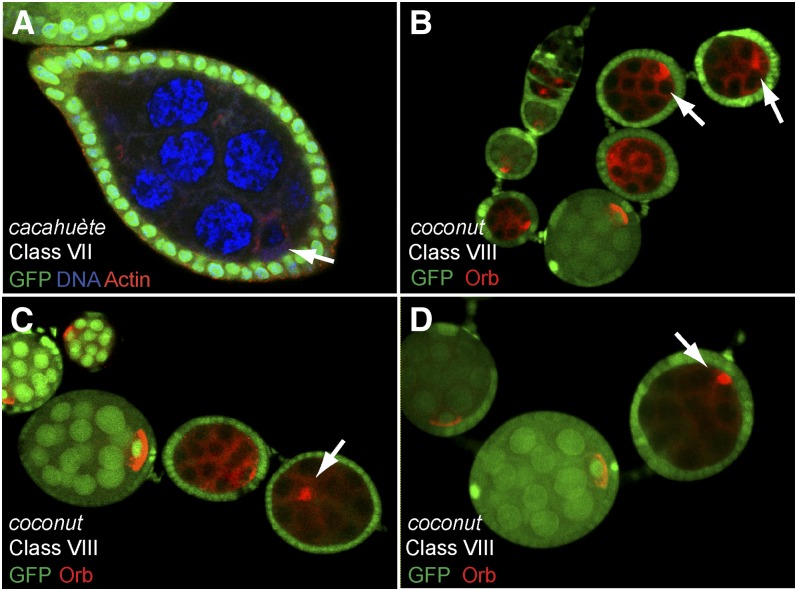
Phenotypic description of the mutants of Classes VII, VIII. (A−D) Mutant germline clones labeled by the lack of GFP (green). (A) *cacahuète* mutant germline clone stained for actin (red) and DAPI (blue). Germline clones contain 15 nurse cells and one tiny oocyte (arrow). Mutant egg chambers have thus the shape of lozenge. (B−D) *coconut* mutant germline clones stop their development around stages 4-5 (arrows). (B, C) Orb protein (red) is correctly restricted to one cell, but forms a round sphere and never makes a tight crescent along the posterior cortex (arrows). (D) In some cases, Orb (red) and the oocyte are mislocalized in the egg chamber (arrow).

During the first round of screen, we identified one line, called *cacahuète*, which gave rise to lozenge shape egg chambers at a high frequency. In contrast to Class I mutants, we found during the secondary screen that the oocyte was well determined and polarized. The size of the oocyte, however, appeared very small compared to the nurse cells and wild type oocytes (Figure 8, A and A′). This specific failure to grow from the oocyte is reminiscent of phenotypes induced by mutations in *Src64*, *Tec29* or *pendulin* (Dodson *et al.* 1998; Roulier *et al.* 1998; Gorjanacz *et al.* 2002). In these mutant egg chambers, ring canals are reduced in size and often degenerate. As a consequence, nurse cells-to-oocyte transport is impaired and the oocyte growth is dramatically affected. A staining for the actin cytoskeleton showed that the ring canals did not degenerate, but appeared smaller than wild type.

### Class VIII: Mutant affecting Orb localization to the posterior cortex

*coconut* is the only allele in this class, which is characterized by an arrest of oogenesis at stage 4−5, which is later than most of our mutants. Centrosomes and Orb were correctly restricted to a single cell and had migrated or were migrating around the oocyte nucleus (Figure 8B). However, Orb never reached the posterior cortex of the oocyte to form a tight crescent. Instead, Orb formed a round sphere (probably the Balbiani body) on the side of the oocyte nucleus (Figure 8C). At later stages, Orb and the oocyte itself became mislocalized within the egg chamber (Figure 8D). The *coconut* allele is sub-lethal and some male and female escapers were viable but sterile. In homozygous mutant females, oogenesis was stopped a little earlier than in germline clones, but the phenotype was nearly identical, indicating that the effect of the mutation is mostly germline-dependent (data not shown). We mapped the mutation to a narrow region, 21B5-21B8, as *coconut* is fully lethal over several overlapping deficiencies.

### *nutmeg* mutations disrupts CG11188, which encodes a Drosophila homolog of Che-1/AATF

**Figure 9:**
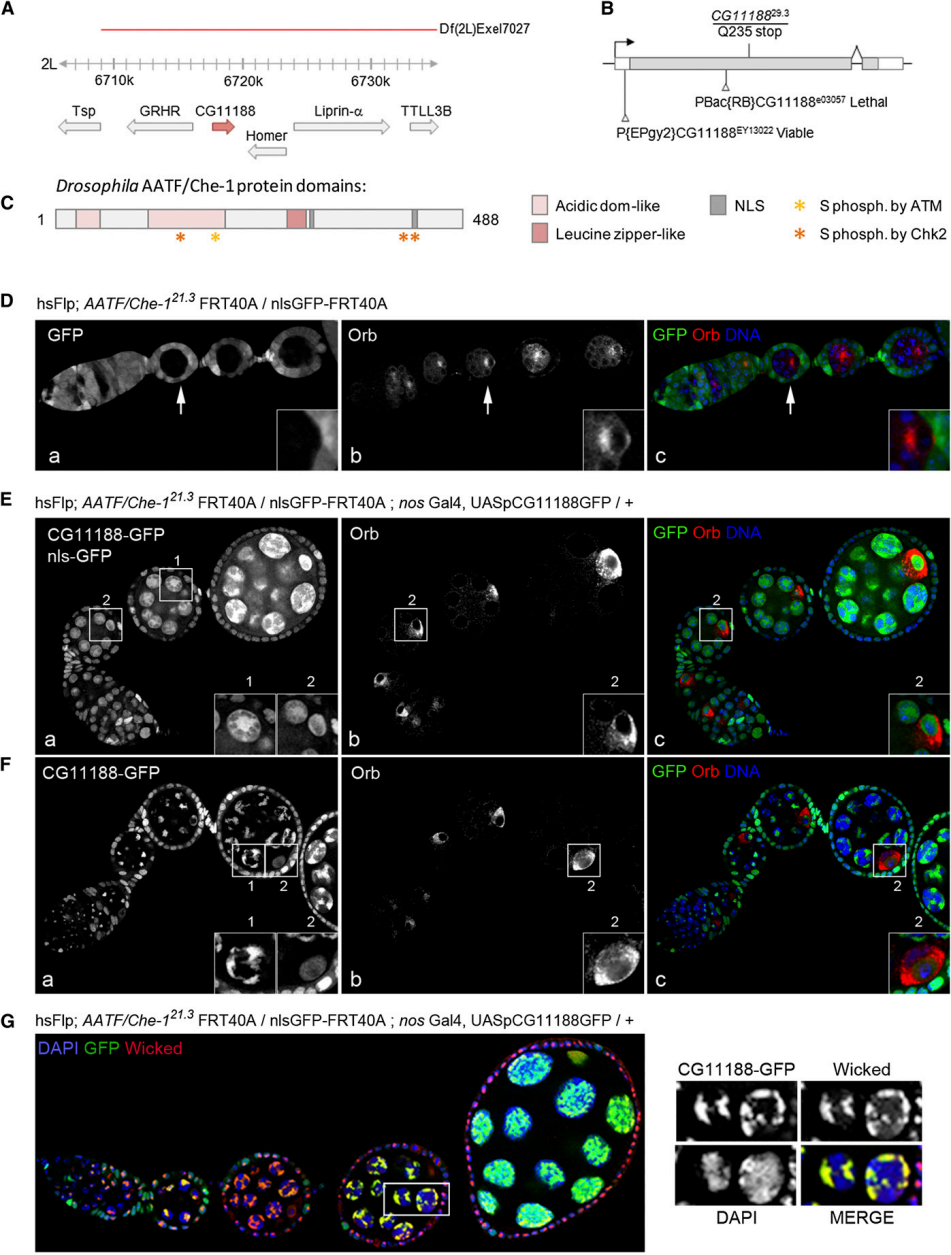
CG11188 corresponds to Drosophila AATF/Che-1, a nucleolar protein involved in oogenesis. (A) Schematic representation of *CG11188* (red arrow) genomic region and genes located nearby (gray arrows) in 2L chromosome. The deficiency leading to lethality when crossed with mutated alleles of *CG11188* is shown by a red line. (B) Schematic representation of *CG11188* gene. Black arrow shows the sense of transcription. White boxes correspond to 3′UTR and 5′UTR. Gray box corresponds to the coding sequence. *CG11188^29.3^* allele codes for a truncated protein (Q235stop) and available transposon insertions are shown. (C) Drosophila AATF/Che-1 conserved protein domains are depicted: acidic domains are in light pink; leucine zipper is in red; nuclear localization signals (NLS) are in gray; putative serine (S) phosphorylated by ataxia telengiectasia (ATM) or by checkpoint kinase 2 (Chk2) are indicated by a yellow or an orange star, respectively. (D) Mosaic ovarioles dissected from hsFLP; *AATF/Che-1^21.3^* FRT40A flies were fixed and stained for Orb (red) and DAPI (blue). Mutant egg chambers can be easily detected by the absence of NLS-GFP. Magnification shows Orb mislocalized to the anterior of the oocyte in a mutant arrested chamber indicated by a white arrow. (E−F) Same flies as in D overexpressing CG11188-GFP were dissected. Ovarioles were fixed and stained for Orb (red) and for DAPI (blue). (E) WT egg chambers express nls-GFP and CG11188-GFP. (F) Mutant chambers only express CG11188-GFP. Magnifications correspond to: 1, GFP signal in a nurse cell. 2, GFP and Orb localization in the oocyte. CG11188-GFP rescues Orb localization and egg chamber growth. (G) Same flies as in E−F were stained for the nucleolar protein Wicked. Magnifications correspond to nurse cells in which nucleolar GFP and wicked signals can be discriminated from nuclear DAPI signal. CG11188-GFP and Wicked perfectly colocalized in the germline.

Because the two alleles of *nutmeg* showed a very penetrant Class II phenotype, we decided to identify the gene disrupted by these mutations. We found that both alleles were lethal over Df(2L)Exel7027 in region 27A1 (Figure 9A). We then tested complementation with mutations in the region and found that both alleles were lethal over insertion PBac(RB)CG11188^e03057^ and were female sterile over insertion P(EPgy2)CG11188^EY13022^. Both transposons are inserted in the *CG11188* locus in the first exon and 5′UTR respectively (Figure 9B). We thus sequenced the CG11188 locus of *nutmeg* mutant homozygous larvae and found that the *nutmeg^29.3^* mutation is a nonsense mutation at amino acid Q235, which leads to the translation of a truncated protein (Figure 9B). We were not able to find a meaningful base pair modification in the *CG11188* locus for the second allele *nutmeg^21.3^*. However, the phenotypes of both alleles during oogenesis are fully rescued by the expression of *CG11188* cDNA (Figure 9, E and F). In addition, germline clones homozygous mutant for PBac(RB)CG11188^e03057^ displayed identical phenotypes to *nutmeg* alleles (data not shown). We thus concluded that *nutmeg^29.3^* and *nutmeg^21.3^* are two alleles of CG11188, a gene that has not been studied before in Drosophila.

A BLAST homology search analysis revealed that CG11188 is the Drosophila homolog of human Che-1, rat anti-apoptotic transcription factor (AATF), and mouse Traube, named Che-1/AATF hereafter (Page *et al.* 1999; Fanciulli *et al.* 2000; Thomas *et al.* 2000). Like its homolog, it contains two acidic domains, one leucine zipper domain, two NLS sequences and predicted phosphorylation sites by ATM and Chk-2 (Figure 9C and Supporting Information, Figure S1). Human Che-1 was originally identified as a RNA Polymerase II binding protein involved in the transcriptional regulation of E2F target genes (Fanciulli *et al.* 2000). It has now been shown that Che-1 is an antiapoptotic factor that links transcriptional regulation, cell cycle control, and DNA damage response (Floridi and Fanciulli 2007; Passananti *et al.* 2007). Surprisingly, the mouse homolog Traube was shown to localize in the nucleolus, a subnuclear compartment predominantly involved in ribosome synthesis (Thomas *et al.* 2000). We investigated the localization of Drosophila Che-1/AATF by analyzing the localization of a CG11188 cDNA tagged with a GFP at the C-terminus and expressed in germline cells homozygous mutant for the endogenous Che-1/AATF to limit overexpression. This construct is functional as it rescues egg chamber growth and Orb localization to the posterior of the oocyte (Figure 9, E and F). Consistent with mouse Traube localization, we found that Che-1/AATF was restricted to the nucleolus of germline cells as it perfectly colocalized with Wicked, a recently identified nucleolar protein [Figure 9G (Fichelson *et al.* 2009)].

**Figure 10:**
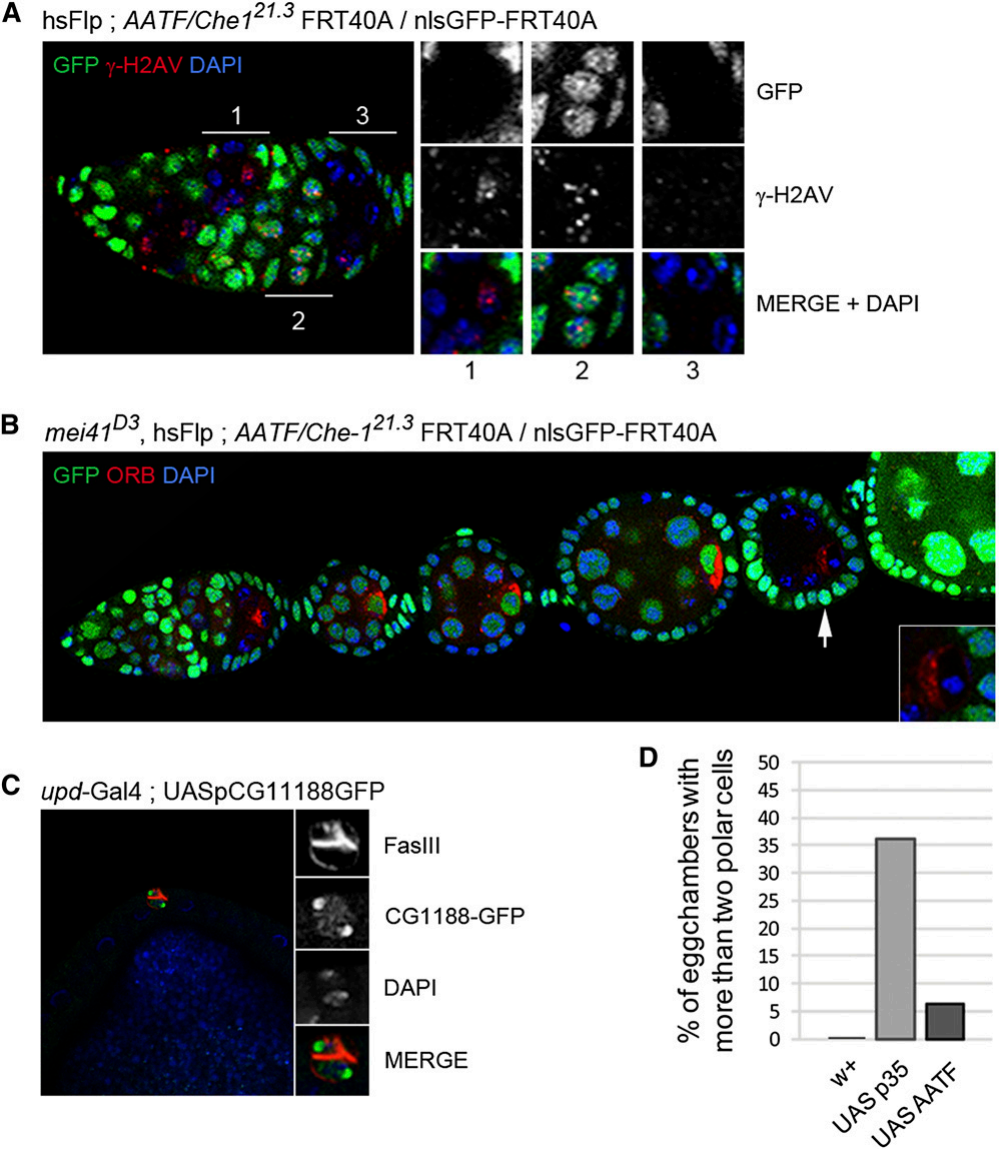
Drosophila AATF/Che-1 is not involved in meiotic DSB repair dependent on Mei-41 and plays a moderate antiapoptotic role in polar cells. (A) Mosaic ovariole dissected from hsFLP; *AATF/Che-1^21.3^* FRT40A/nlsGFP-FRT40A flies were fixed and stained for γ-H2AV to detect DSB foci (red) and DAPI (blue). Mutant chambers are distinguished by the absence of nls-GFP (green). Magnifications correspond to: a clonal (1) and a WT (2) chamber in region 2 of the germarium; (3) a clonal chamber in region 3 of the germarium. (B) Ovarioles from the same flies as in A also mutated for *mei-41^D3^* were dissected, fixed, and stained for Orb (red) and for DAPI (blue). Mutant chambers are distinguished by the absence of NLS-GFP (green). Magnification corresponds to the indicated mutated chamber (white arrow) where Orb remains to the anterior of the oocyte. (C) Ovarioles from flies overexpressing CG11188-GFP (green) in polar cells under the control of *unpaired* (*upd*-Gal4;UASp::CG11188-GFP) were dissected, fixed and stained for Fas-III to detect polar cells (red) and DAPI (blue). (D) The percentage of egg chambers with more than two polar cells was calculated in ovarioles from flies overexpressing CG11188 (UAS::AATF) or the caspase inhibitor p35 (UAS::p35).

In response to DNA damage, Che-1 was shown to be phosphorylated by the checkpoint kinase ATM and its effector Chk2 (Bruno *et al.* 2006; Halazonetis and Bartek 2006). These multiple phosphorylations stabilize Che-1/AATF, which then activates the transcription of p21Waf1 and p53. The increased levels of p21 and p53 are required to maintain the G2 arrest in response to DNA damage. Endogenous DNA double-strand breaks (DSBs) are generated during prophase I of meiosis, which takes place in region 2a of the germarium (McKim *et al.* 2002). It was recently shown that these meiotic DSBs induce the activation of the p53 regulatory network (Lu *et al.* 2010). In addition, a failure to repair meiotic DSBs or premeiotic DNA damages leads to developmental defects during oogenesis (Ghabrial and Schüpbach 1999; Narbonne-Reveau and Lilly 2009). We thus tested whether the early arrest in egg chamber development mutant for *Che-1/AATF* could be due to an inappropriate response to DNA damage occurring in region 2a of the germarium. We first analyzed the levels and timing of appearance and disappearance of DSBs marked by the phosphorylation of a histone variant γH2Av [γH2AX in vertebrates (Mehrotra and McKim 2006)]. We found that the number and timing of appearance of DSB foci was similar between *Che-1/AATF* mutant and wild type cysts (compare inset 1 and 2 on Figure 10A). In addition, DSBs foci disappeared in region 3 in *Che-1/AATF* mutant germline cyst (inset 3, Figure 10A) as in wild type (Mehrotra and McKim 2006). However, activation of the meiotic checkpoint is not always associated with a detectable increase of γH2Av phosphorylation (K. McKim, personal communication). Furthermore, it remains unknown whether Che-1/AATF acts only downstream of ATM/Chk-2 in a simple linear cascade or whether it is associated with other pathways in response to DNA damage in different species. To test whether the activation of the meiotic checkpoint was responsible for the early arrest of *Che-1/AATF* mutant egg chambers, we induced *Che-1/AATF* homozygous mutant germline clones in a background homozygous mutant for *mei41*, the Drosophila ATR homolog and the main meiotic checkpoint kinase. We found that egg chambers double mutant for *mei41* and *Che-1/AATF* were indistinguishable from single *Che-1/AATF* mutant cyst (n = 27; Figure 10B). We thus concluded that the early arrest in *Che-1/AATF* mutant egg chamber was not dependent on *mei41* activity.

Che-1/AATF antiapoptotic activity was demonstrated in several cell types by its activation of antiapoptotic factors such as XIAP and its binding and inhibition of proapoptotic factors such as Dlk, Par-4, and NRAGE (Passananti *et al.* 2007). To test Che-1/AATF antiapoptotic activity *in vivo*, we overexpressed *CG11188* cDNA tagged with GFP in precursors of polar cells in the follicular epithelium. Polar cells are pairs of specific follicle cells localized at the two poles of each egg chambers. The two polar cells are selected from a group of three to five pre-polar cells by an apoptosis-dependent mechanism (Besse and Pret 2003). We found that in wild-type egg chambers 100% of polar cells clusters are made of two cells by stage 6, whereas in cells overexpressing Che-1/AATF 6% had more than two cells (n = 80; Figure 10, C and D). This is result should be compared with 35% of clusters with more than two cells when we overexpressed p35 (n = 61; Figure 10D), the strongest inhibitor of apoptosis in this system and in our hands. We concluded that Drosophila Che-1/AATF can inhibit, to a moderate extent, endogenous apoptosis *in vivo*.

## Discussion

In this study, we report the results of a clonal screen designed to isolate mutants on the left arm of chromosome two, in which the early steps of Drosophila oogenesis are defective. We screened 3257 mutagenized lines and identified nine multiallelic complementation groups. We also kept several single alleles exhibiting interesting phenotypes. These complementation groups include the previously described gene *Bicaudal-D*, which is involved in oocyte determination, and known genes such as *Gmd*, the metalloprotease *Kuzbanian*, the tumor suppressor gene *l(2)gl*, the nuclear export factor *Tho2*, and the elongation factor *eIF4A*. Only five of our mutants are homozygous viable, whereas the rest of our alleles are homozygous lethal and thus would not have been isolated in a traditional female sterile screen. Moreover, the majority of these mutants exhibit developmental defects prior to stage 6 of oogenesis, indicating that they would not have been recovered in a clonal screen using the OvoD system.

### Saturation

Although we hit several expected genes in our screen, we also missed some of them, indicating that our screen did not reach saturation. For example, we did not isolate new alleles of *missing oocyte* (*mio*), which is required to maintain the oocyte in meiosis (Iida and Lilly 2004), nor did we find novel alleles of *string-of-pearls* (*sop*), which encodes the Drosophila ribosomal protein S2 (Cramton and Laski 1994). Another indication that our screen did not reach saturation is that, after the exclusion of the 19 mutants genetically interacting in Class III, 29 mutants (40%) remain single alleles. One possible explanation is that our screening procedure selected only for a specific window of allele strength. Strong alleles of genes involved in general cellular functions would for example lead to cell death without forming a 16-cells cyst and would not have been kept in our screen. On the other hand, partial loss-of-function of genes involved at several stages of oogenesis may also not have been selected as they would not cause penetrant early arrests of oogenesis. For instance, only the strongest alleles of *par-1* stop oogenesis early (Shulman *et al.* 2000; Huynh *et al.* 2001b), whereas hypomorphic combinations are viable and lead to a late grandchildless phenotype (Shulman *et al.* 2000; Tomancak *et al.* 2000). In agreement, the two alleles of *BicD* identified in our screen are indistinguishable from *BicD^r5^*, a protein-null version of *BicD* (Suter *et al.* 1989; Wharton and Struhl 1989; Ran *et al.* 1994). We thus have likely missed some hypomophic alleles of the genes identified in our screen as they do not affect visibly early oogenesis.

### Class II: Mutant arresting oogenesis before oocyte repolarization

The main category of mutants identified in our screen is Class II, because almost half of the lines (32/71) are in this phenotypic group. Half of these mutants (17/32) fall into six multiallelic complementation groups. Three genes were identified molecularly: *tho2*, *eIF4A*, and *CG11188*.

Tho2 is a component of the THO complex identified in yeast and plays an essential role in nuclear export of mRNAs (Chavez *et al.* 2000). The THO complex recruits general export factors such as NFX-1 and UAP56 while mRNAs are still being transcribed and forms RNP complexes (RiboNucleoProteins) ready to be exported (Köhler and Hurt 2007). It is possible that defects in this general function could lead to an early arrest of oogenesis without being related to the polarization of the oocyte. However, it was shown in Drosophila S2 cells that the THO complex could be dispensable for the export of the majority of mRNAs, suggesting that this complex could play a different, albeit unknown, function in Drosophila cells (Rehwinkel *et al.* 2004). Interestingly, homologs of both NFX-1 and UAP56 are also required during Drosophila oogenesis. In the absence of *small bristles* (*sbr*), the ortholog of human TAP/NFX-1 and yeast Mex67, egg chambers rapidly degenerate once in the vitellarium (Wilkie *et al.* 2001). *sbr* and *tho2* share an additional phenotype as both mutants induce the formation of small bristles. Although essential for mRNA nuclear export, UAP56 is also required for post-transcriptional modifications and localization of mRNA in the oocyte cytoplasm at later stages of oogenesis (Meignin and Davis 2008). Furthermore, a recent study found that *Tho2* homolog in tobacco is a microtubule associated protein. In interphase, the cytoplasmic fraction of Tho2 localizes along cortical microtubules, while during mitosis it localizes on the mitotic spindle (Hamada *et al.* 2009). A RNAi screen performed in S2 cells also identified Tho2 as a regulator of mitosis and spindle formation (Somma *et al.* 2008). Because MTs are essential for the early polarization of the oocyte, we thus cannot exclude a cytoplasmic function of Tho2 during this process.

*eIF4a* encodes a DEAD box ATP-dependent RNA helicase, which is essential for translation initiation and has a well-described function in cellular growth and cell division. More recently, *eiIF4a* was identified as a new component of the polar granules in the germ plasm at the posterior of the oocyte (Thomson *et al.* 2008). The function of *eIF4a* during Drosophila oogenesis remains, however, unknown. Vasa, another DEAD box ATP-dependent RNA helicase localizing also in the polar granules, is known to be essential at multiple stages of oogenesis. Analysis of a null mutation of *vasa* revealed that the protein is required for the germline cyst development and the early oocyte differentiation, in addition to its well-described function in posterior embryonic patterning and pole cell specification (Styhler *et al.* 1998; Tomancak *et al.* 1998). *eIF4a* could be a partner of Vasa during the early stages of oogenesis.

### Egg chambers growth defects and the insulin pathway

Our second most important group (25/71) is made of mutants in which egg chambers fail to grow, although the oocyte is correctly determined and polarized. A total of 19 of these mutants strongly interact genetically between each other, and 17 of them also interact genetically with *chico*, a direct substrate of the Insulin receptor. In contrast, we did not detect genetic interaction with the ribosome biogenesis pathway. In addition, only one out of 25 class III mutants interact genetically with *p60*, none with *dp110*. We consider it unlikely that all 17 mutants are alleles of *chico* for several reasons: (1) *chico* locus is small (4 kb) and our mutagenized stock was isogenized several times during the course of the screen; and (2) at least three of our alleles did not show any changes in the coding sequence of *chico*.

Chico is known to be required for germline cyst growth and it was further shown that the binding of p60, but not of Grb2/Drk to Chico is also required for vitellogenic stages (Böhni *et al.* 1999; Richard *et al.* 2005; Hsu *et al.* 2008). In addition, we showed that germline clones mutant for *p60* and *dp110* were indistinguishable from class III mutants. These results suggest that the insulin pathway is mainly mediated by dp110 and its adaptor p60, during oogenesis. However, most of our mutant lines are not alleles of *chico* and do not interact genetically with *p60* nor *dp110*, suggesting that a pathway parallel to dp110 may exist downstream of the insulin receptor during oogenesis.

### *coconut* is a female sterile mutation required for Orb localization at the posterior cortex

The localization of Orb as a sphere on the side of the oocyte nucleus is a rare phenotype. It is most similar to the localization of *oskar* mRNA and BicD::GFP described in *maelstrom* (*mael*) null mutant oocytes. *mael* mutant flies also are viable and sterile, although oogenesis proceeds further as dorsoventral axis defects become apparent later during oogenesis. *maelstrom* is some time classified as a spindle-class gene (Clegg *et al.* 1997, 2001; Findley *et al.* 2003). In contrast, oogenesis stops at stage 4−5 in *coconut* mutant flies. In addition, *coconut* is fully lethal over several deficiencies indicating that it is a hypomorphic allele. Maelstrom localizes in a cytoplasmic structure called *nuage* and in the nucleus of germ cells. In mouse and flies, it represses retrotransposons during spermatogenesis via the piRNA pathway (Cook *et al.* 2004; Soper *et al.* 2008). A recent study further showed that *mael* can repress miRNA transcription also during fly spermatogenesis (Pek *et al.* 2009). We have localized the *coconut* mutation to an 80-kb region in 21B5, but there is no obvious candidate gene in this region, which would fit with a function in the piRNA or miRNA pathways.

### *CG11188* encodes a Drosophila homolog of the antiapoptotic factor Che-1/AATF

We found that the two alleles of *nutmeg* disrupt the novel locus *CG11188*. We showed that CG11188 is the Drosophila homolog of Che-1/AATF, a transcription factor involved in the control of the cell cycle, DNA damage response, and regulation of apoptosis (Floridi and Fanciulli 2007; Passananti *et al.* 2007). Consistent with this central role in DNA damage response, it was shown that inhibition of Che-1/AATF strongly enhanced the cytotoxicity of anti-cancer drugs, suggesting Che-1 as a possible therapeutic target to increase the efficiency of DNA damaging drugs (Bruno *et al.* 2006; Halazonetis and Bartek 2006). During Drosophila oogenesis, we could not link Che-1/AATF to the presence of endogenous DSBs in the early phases of meiosis. We did not detect an increase or perdurance of DSBs and the arrest of *Che-1/AATF* mutant egg chambers did not depend on the activity of Mei41. However, it remains possible that there are DSBs that we did not detect by antibody staining and that could in addition activate a Mei41-independent checkpoint as suggested by several reports (Barbosa *et al.* 2007).

We demonstrated that Drosophila Che-1/AATF can have a modest antiapoptotic activity when overexpressed in the polar cells precursors. Could this antiapoptotic function be responsible for the early arrest in *Che-1/AATF* mutant egg chambers? Endogenous apoptosis of germ cells have been characterized in Drosophila, and three stages have been distinguished (McCall 2004): (1) early in region 2 of the germarium; (2) at mid-oogenesis; (3) during the late stages of oogenesis when nurse cells dump their cytoplasm into the oocyte. In contrast, germline clones mutant for *Che-1/AATF* are consistently arrested at stage 2−3 (Figure 9D) and we did not detect a significant increase in cell death in germ cells mutant for *Che-1/AATF* (data not shown). The antiapoptotic function of Che-1/AATF thus doesn’t seem to be the main reason for the early arrest during oogenesis.

An alternative cause of arrest could be the proposed function of the mouse homolog, Traube, in ribosomes biogenesis (Thomas *et al.* 2000). Indeed, mouse embryos mutant for Traube arrest their development at an early stage (E2.5) and analysis of these mutant embryos by electron microscopy revealed a visible decrease in ribosomes and rough endoplasmic reticulum (Thomas *et al.* 2000). Interestingly, mutants affecting the synthesis of ribosomes in Drosophila, such as *string-of-pearl* (*sop*) or *wicked* (*wkd*), arrest the development of egg chambers at the same stage as *Che-1/AATF*, *i.e.*, before the translocation of Orb and the centrosomes to the posterior cortex of the oocyte (Cramton and Laski 1994; Fichelson *et al.* 2009). Moreover, this stage 2−3 of oogenesis also corresponds to the start of a massive growth of the egg chambers. It is thus possible that, among all Che-1/AATF functions, the main cause of arrest of mutant egg chambers is a failure to synthesize ribosomes needed to trigger this growth. The molecular and biochemical function of Che-1/AATF in ribosomes biogenesis remain however completely unknown and is still to be characterized.

### Conclusion

Our screen has generated a number of mutant lines affecting several aspects of Drosophila early oogenesis. We hope that our collection of mutants on chromosome 2L together with a previously generated collection on chromosome 3R (Morris *et al.* 2003), can be used as a starting point for further investigations at the molecular level.

## Supplementary Material

Supporting Information
