# Supplementary material for: A Mosaic Genetic Screen for Genes Involved in the Early Steps of Drosophila Oogenesis
Source: G3 (Bethesda). 2013 Mar 1;3(3):409–25. doi: 10.1534/g3.112.004747 (PMC3583450; doi:10.1534/g3.112.004747)
Supplement: Supporting Information [file supp_3_3_409__index.html]

Supporting Information 

# A Mosaic Genetic Screen for Genes Involved in the Early Steps of Drosophila Oogenesis

## Supporting Information for Jagut *et al.*, 2013

**Files in this Data Supplement:**

- Figure S1 - Sequences comparison of CG11188 and Che-1/AATF related proteins (PDF, 550 KB)
