# Supplementary material for: A Mosaic Genetic Screen for Genes Involved in the Early Steps of Drosophila Oogenesis
Source: G3 (Bethesda). 2013 Mar 1;3(3):409–25. doi: 10.1534/g3.112.004747 (PMC3583450; doi:10.1534/g3.112.004747)
Supplement: Supporting Information [file supp_3.3.409_004747SI.pdf]

G3·Genes | Genomes | Genetics

A Mosaic Genetic Screen for Genes Involved in the Early Steps of Drosophila

Marlène Jagut[1, 2], Ludivine Mihaila-Bodart[1, 2], Anahi Molla-Herman[2], Marie-Françoise Alin[1], Jean-Antoine Lepesant[1] and Jean-René Huynh[1, 2, 3]

[1]Institut Jacques Monod, CNRS-Universite Paris Diderot, Bât. Buffon - 15 rue Hélène Brion 75205 Paris cedex 13, France.

[2] Institut Curie, Department of Genetics and Developmental Biology (CNRS-UMR3215, Inserm-U934), 26 rue d'Ulm, 75248 Paris, Cedex 05, France

[3] Author for correspondence, email: jean-rene.huynh@curie.fr

DOI: 10.1534/g3.112.004747

CG11188 - Drosophila melanogaster Alignments
mercredi 9 juin 2010 16:46

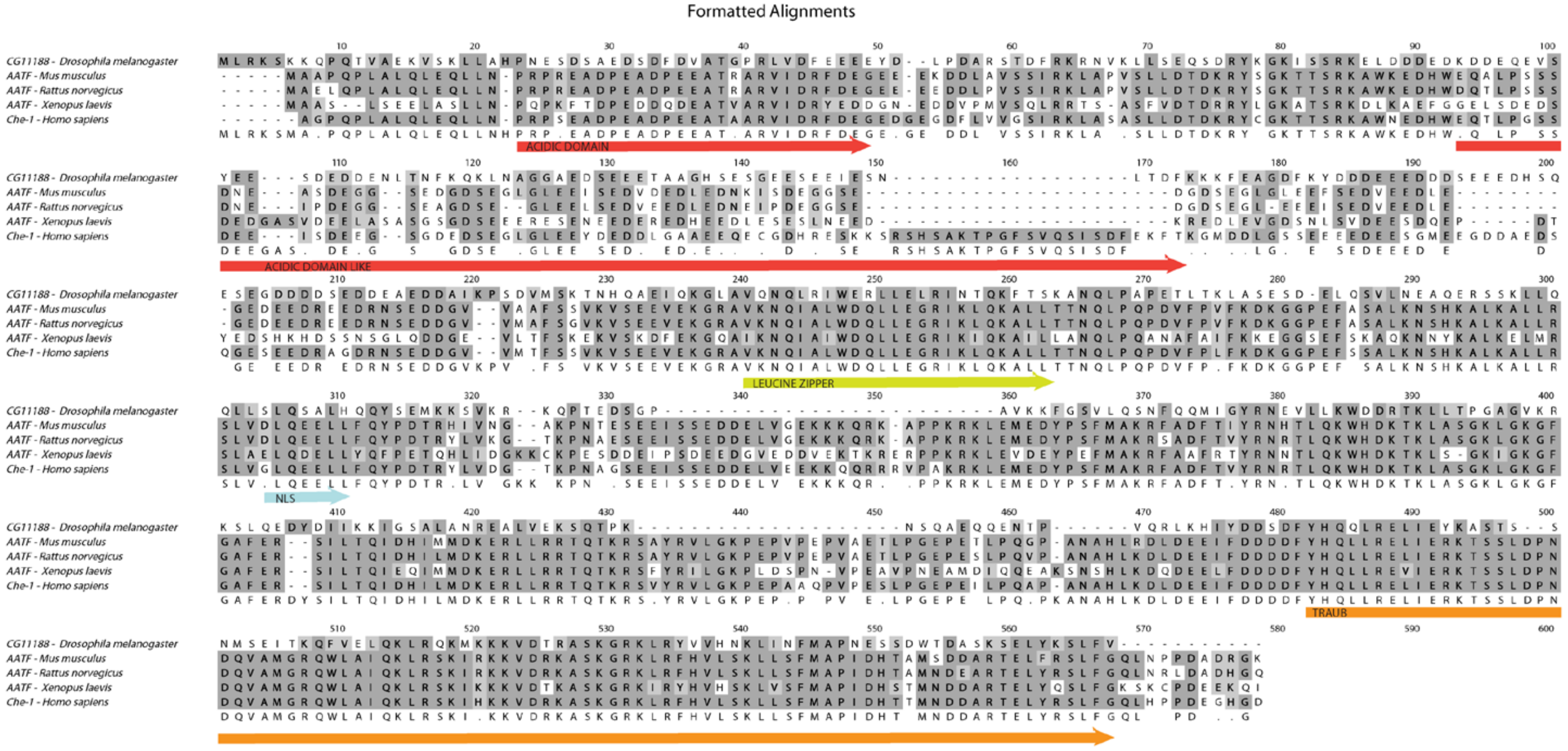

Figure S1 Sequences comparison of CG11188 and Che-1/AATF related proteins. Amino acid sequence comparison of CG11188 of *Drosophila melanogaster* (Accession No. AAF52427), AATF of *Mus musculus* (Accession No. NP_062790), AATF of *Rattus norvegicus* (Accession No. AAH78769), Che-1 of *Homo sapiens* (Accession No. AAH00591) and AATF of *Xenopus laevis* (Accession No. NP_001167477). Identical amino acid residues are shown strongly shaded gray in all sequences in red, similar residues are lightly shaded gray, and different residues are not shaded.
